# A Study on the Willingness and Factors Influencing the Digital Upgrade of Rural E-Commerce

**DOI:** 10.3390/bs13020095

**Published:** 2023-01-23

**Authors:** Yuncheng Wang, Zhongming Zhang

**Affiliations:** College of Economics and Management, China Jiliang University, Hangzhou 310018, China

**Keywords:** rural e-commerce, theory of planned behavior, structural equation modeling, willingness to digitally upgrade

## Abstract

The current problems of the low digital upgrading of rural e-commerce, single marketing method and high homogeneous competition will seriously hinder the development of rural e-commerce and rural revitalization. Therefore, finding how to guide the digital upgrading of rural e-commerce will be a key step in achieving rural revitalization and common prosperity. Based on 1387 Zhejiang rural e-commerce digital upgrading questionnaires, this paper analyzes willingness regarding rural e-commerce digital upgrading and its influencing factors using planned behavior theory and structural equation modeling. The findings show that (1) behavioral attitudes are the main influencing factors of upgrading willingness, and subjective norms are secondary influencing factors; (2) in terms of the behavioral attitudes of rural e-merchants, the greater the proportion of their online sales or the higher the proportion of online income, the better behavioral attitudes formed on this basis can promote the willingness to digitally upgrade rural e-merchants; (3) in terms of subjective norms, when rural e-merchant subjects perceive that the increased local participation in e-merchant association is too large or online income tends to saturate the total income, the more subjective norms can influence their willingness to digitally upgrade.

## 1. Introduction

Since 2014, rural e-commerce has been written into the No. 1 document of the Central Government for nine consecutive years, and the No. 1 document of 2022 clearly points out that rural e-commerce is the focus of the integrated development of one, two and three industries, the implementation of the “digital business prosperous agriculture” project, and the promotion of e-commerce in rural areas. As a product of the integration of the Internet with modern agriculture, rural modernization and informatization, rural e-commerce has become a new driving force to promote rural revitalization and is a new carrier of common prosperity [1]. However, as China’s rural e-commerce has gradually matured, a series of problems have come to the fore. This includes both the problems of rural e-commerce development, such as the single-channel model, serious homogenization of products, and non-uniform production standards, and the impact of external policies, economic environment, and technology level. All these present new challenges for the further development of rural e-commerce.

In response to the dilemma faced by rural e-commerce, the digitalization of the countryside has brought a new round of development opportunities. In January 2022, the State Council issued the “Fourteenth Five-Year Plan” for the development of the digital economy to further enhance the digitalization of agricultural sales and convergence of the digital empowerment of rural e-commerce. At the same time, the National Rural Revitalization Bureau and another ten ministries jointly issued the “Digital Countryside Development Action Plan (2022–2025)” to implement the digital optimization and upgrading of rural e-commerce. The central government promulgated the “14th Five-Year Plan” to promote agricultural and rural modernization plans for the development of rural e-commerce deployment. This aimed to promote the digital transformation of rural e-commerce and implement “digital business to promote agriculture”. The above policies show that the digitalization of rural e-commerce is a key focus point and important to the rural digital economy. However, compared with the digitalization of urban e-commerce strides, the digitalization of rural e-commerce lags behind compared to the traditional platform model. The realization of digital transformation and upgrading of rural e-commerce has become an important issue in the digital countryside strategy. The existing studies reveal three motivations for the adoption of ICT in rural communities: extrinsic, intrinsic, and mixed motivations. The results further suggest that ICT availability, support from farmer groups and families, and inspiration, joy, hope and trust are motivating factors for successful ICT acceptance and continuation [2]. ICT also plays a positive role in improving e-governance in rural areas [3] and is seen as a key means to address the lack of information, ineffective communication and emotional disconnect in rural areas. Most studies on ICT can be categorized as top-down development approaches, usually theoretical studies or case studies using qualitative methods [4]. Although the factors influencing ICT adoption in rural communities are closely related to users’ behavioral motivations, a systematic categorization of these factors and a distinction between the magnitude of the impact of different factors is lacking. In addition to this, the digitization of agricultural e-commerce is also closely related to farmers’ online purchasing behavior, which is determined, to some extent, by their willingness to use it [5]. In view of this, this paper constructs a model based on the theory of planned behavior and explores the willingness of rural e-merchants to digitize and upgrade and the influencing factors to provide theoretical guidance and practical experience for the digitization of rural e-merchants.

## 2. Materials and Methods

### 2.1. Theoretical Basis

The theory of planned behavior was developed by Fishbein and Icek Ajzen [6] and is a further extension of the theory of rational behavior. The theory argues that human behavior is not 100% voluntary but under control, thus adding a new variable of perceived behavioral control to the theory of rational behavior. In the theory of planned behavior, individual behavior depends, to some extent, on willingness, which is determined by three key variables: behavioral attitudes, subjective norms, and perceptual behavioral control. To further clarify this theory, Ajzen interpreted the three key variables of the theory of planned behavior specifically as the strength of behavioral beliefs, behavioral outcome assessment, normative beliefs, motivation to conform, control beliefs, and perceptual strength. The theory is mainly applied in studies on pro-environmental behavior, food waste behavior, entrepreneurial behavior, and public behavior in the use of personal care products and has yielded good experimental results that have advanced the study of behavioral motivation in these areas [7,8,9,10].

### 2.2. Research Hypothesis

**Figure 1:**
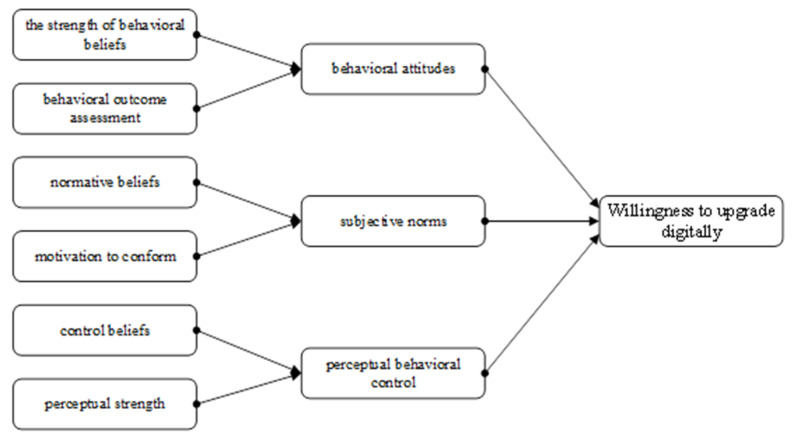
Hypothetical model of factors influencing willingness to digitally upgrade rural e-commerce.

In this study, based on the theory of planned behavior, the hypothesis that behavioral attitudes, subjective norms and perceived behavioral control affect willingness to digitally upgrade rural e-commerce is proposed, along with the fact that these three factors are also influenced by the strength of behavioral beliefs, behavioral outcome assessment, rural e-commerce households’ normative beliefs, compliance motivation, control beliefs and perceived strength. This leads to the construction of a hypothetical model of the willingness to digitally upgrade rural e-merchants, as shown in Figure 1.

(1) Behavioral attitudes and willingness to digitally upgrade rural e-merchants. According to the theory of planned behavior, behavioral attitudes refer to the positive or negative judgments of rural e-commerce households regarding digital upgrading behavior. The more positive the behavioral attitudes of rural e-commerce households, the stronger the willingness to digitally upgrade, which is determined by the strength of their behavioral beliefs and the assessment of behavioral outcomes.

From the theory of planned behavior, it is clear that the strength of behavioral beliefs is an important determinant of the behavioral attitudes of rural e-commerce households. In the model of rural e-commerce households’ willingness to digitally upgrade, the strength of behavioral beliefs refers to e-commerce households’ psychological expectation that the digital upgrade will bring increased income from e-commerce. The behavioral attitude is stronger when their psychological expectation of a higher income from digital upgrade is higher and vice versa. The findings show that the strength of rural e-commerce households’ behavioral beliefs is firstly reflected in the recent average annual income: when farmers’ average annual income is lower, their adaptability is worse [11], and their hope of receiving cash compensation from the government is greater [12,13]; therefore, the strength of their behavioral beliefs is lower. The strength of rural e-commerce households’ behavioral beliefs is also reflected in supply, access and elicitation. Studies have shown that rural e-commerce households’ degree of dependence on the supplier will influence their decision-making behavior [14,15] and affect the strength of their behavioral beliefs. In addition, in the process of digital upgrading, rural e-commerce households will gradually increase their dependence on the platform and lose their autonomy [16], lack ways to attract flows from third-party platforms, and have low motivation to digitally upgrade, which will also reduce the strength of their behavioral beliefs.

In addition to the strength of their behavioral beliefs, e-commerce households’ assessment of behavioral outcomes can also determine the direction of their behavioral attitudes, not only in terms of their assessment of sales volume and revenue growth after operating an e-commerce business, but also in terms of the assessment of outcomes after the digital upgrade. The findings suggest that rural e-commerce operators digitally upgrade to increase sales volume [17,18] and revenue [19,20]. Similarly, during the digital upgrading of e-commerce, digital technology is used to improve distribution efficiency, which expands market sales’ volume [21]. In addition, digital upgrading can also enhance online and offline linkage development and reduce the market risk borne by rural e-merchants, thus promoting the revenue growth and sustainability of e-merchants [22].

Based on the above analysis, it can be seen that the more positive the behavioral attitudes of rural e-commerce households, the stronger their willingness to digitally upgrade, and the better the behavioral belief strength and behavioral outcome assessment of e-commerce households, the more positive their behavioral attitudes. Based on this, this paper proposes the following hypotheses.

**H1**  : *There is a positive influence of behavioral attitudes on willingness to digitally upgrade rural e-commerce*.

**H2**  : *The strength of behavioral beliefs has a positive effect on the behavioral attitudes of rural e-commerce households*.

**H3**  : *Behavioral outcome assessments have a positive impact on the behavioral attitudes of rural e-commerce households*.

(2) Subjective norms and willingness to digitally upgrade rural e-merchants. According to the theory of planned behavior, subjective norms refer to the influence of the support or opposition held by surrounding individuals and organizations that rural e-merchant households perceive after digital upgrading. The subjective norms of rural e-commerce households are influenced by both the normative beliefs of rural e-commerce households and their motivation to conform.

Normative beliefs refer to the extent to which rural e-commerce households believe that the individuals or organizations around them approve of their digital upgrading, specifically including directive norms and exemplary norms [23]. Directive norms mainly refer to government support, and the stronger the government support for rural e-commerce, the more active the participation of e-commerce operators [24,25]. Demonstrative norms mainly refer to the support of e-commerce associations, friends and relatives, neighbors, etc. When farmers perceive a strong supportive attitude from those around them, especially their relatives, regarding changes in their behavior, their subjective norms promote willingness to change behavior [26]. When farmers consider choosing a transaction mode, they will consider the proportion of other e-commerce online transactions in their village [27]. The financial support from the e-commerce association also influences farmers’ normative beliefs [28]. Based on this, it can be inferred that rural e-merchant households may consider the normative requirements of the government, e-merchant associations, friends and relatives, and neighbors when considering whether to digitally upgrade, i.e., how much they support the digital upgrade of rural e-merchant households. Additionally, the support of the government, e-merchant associations, friends and relatives, and neighbors will determine the normative beliefs of e-merchant households.

Obedience motivation refers to the tendency of rural e-commerce households to obey normative beliefs. Individual subjective norms were found to be influenced by the coordination of normative beliefs and obedience motivation in personal intention studies, and the stronger the individual’s normative beliefs, the higher the degree of obedience, and the more positive the individual’s subjective normative influence [29]. The obedience motivation of rural e-commerce households refers to the response to normative beliefs, specifically the choice of e-commerce business type [30], brand creation [31,32], the adoption of marketing methods [33,34], etc. From this, it can be inferred that rural e-commerce households respond to normative beliefs, i.e., compliance motivation.

Based on the above analysis, it can be seen that the more that rural e-commerce households are supported by the external environment, the stronger their subjective norms for digital upgrading and the higher their willingness to upgrade. Based on this, this paper puts forward the following hypotheses.

**H4:**  *Subjective norms have a positive impact on the willingness to digitally upgrade rural e-commerce*.

**H5**  : *Normative beliefs have a positive impact on the subjective norms of rural e-commerce households*.

**H6:**  *Obedience motivation has a positive effect on the subjective norms of rural e-commerce households*.

(3) Perceived behavioral control and willingness to digitally upgrade rural e-commerce. Perceived behavioral control refers to the degree of influence that resources held by rural e-commerce households have on their adoption of a certain behavior. The higher the perceived behavioral control, the greater the individual’s willingness to act and the more likely the behavior will occur, which includes both control beliefs and perceived intensity.

Control beliefs refer to the factors that rural e-commerce households perceive, which may hinder and facilitate their digital upgrading. These mainly include both their own factors and family factors. The results of the study indicate that age, household labor, education level, number of business start-ups and the e-commerce field in which the farmers are engaged affect their decision-making behavior [35]. In addition to this, the level of e-commerce knowledge also has a significant impact on the decision-making behavior of farmers [36,37]. Based on this, it is not difficult to determine that rural e-commerce households’ own factors and environmental factors influence their control beliefs.

Perceived intensity refers to the extent to which rural e-commerce households perceive that environmental factors influence their behavior. It was found that accessibility has a tendency to promote e-commerce operations [38,39], specifically the state of direct road access [40], the proximity of railway stations [41,42] and the proximity of courier points [43]. In addition to this, difficulties such as in the choice of financing model [44] and the creation of a safe atmosphere [45] can also affect the perceptual intensity of rural e-commerce households.

Based on the above analysis, it can be inferred that internal and environmental factors for rural e-commerce households influence their willingness to digitally upgrade. The greater the positive influence of the perceived factors, the stronger the willingness to digitally upgrade. As a result, this paper proposes the following hypotheses.

**H7**  : *Perceptual behavioral control has a positive effect on the willingness to digitally upgrade rural e-commerce*.

**H8**  : *Control beliefs have a positive effect on perceived behavioral control of rural e-commerce*.

**H9:**  *Perceptual intensity has a positive effect on perceptual behavioral control of rural e-commerce.*

## 3. Empirical Research Design

### 3.1. Data Sources

The data used in this paper come from the summer research of 40 college students recruited by the team, and the research area was Zhejiang Province, where the national digital e-commerce demonstration base is concentrated. The research objects for rural e-commerce households were as follows: farmers who operate e-commerce. In order to ensure the authenticity and reliability of the questionnaire data, this study combined the design of the questionnaire with the economy, policies and multiple other factors for each city in Zhejiang Province, while referring to the List of E-commerce Demonstration Villages in Zhejiang Province in 2020, and used the stratified sampling method and random sampling method for sample extraction: (1) The random sampling method was used to extract five cities (Hangzhou, Wenzhou, Jinhua, Quzhou and Shaoxing City) and divide them into five files, with each file adopting a random starting point symmetric equidistant sampling method to draw two districts, presenting a total of ten districts; (2) each district then randomly selected 1–2 townships, with a total of 15 townships; (3) due to the special nature of the survey population, the stratified random sampling method was used for the final step to randomly select 3–4 administrative villages for each of the 15 townships. A total of 50 administrative villages were sampled. The survey period was July–August 2021, and the survey method was one-on-one interviews. The households were confirmed to meet the definition of rural e-commerce households before the interview to ensure the validity of the questionnaire. During the interview process, the researcher filled in the questionnaire in a timely manner according to the responses of the e-commerce households. A total of 1600 questionnaires were collected in the survey; excluding missing values and outliers, 1387 valid questionnaires were obtained, with a valid recovery rate of 86.7%. The basic characteristics of the sample are shown in Table 1.

As shown in the table above, 90% of the surveyed subjects were under 50 years of age, showing that those who operate e-commerce are predominantly young and middle-aged, with 56.8% of young people being under 40 years of age. This indicates that the younger the subject is, the higher their participation in e-commerce operations. In terms of literacy, the percentage of respondents with college education or above was over 50%, which indicates that those who are engaged in e-commerce are relatively highly educated, but there were still a certain number of respondents with a low level of education. In terms of their entrepreneurship and training in e-commerce business, more than half of them had no experience in entrepreneurship and e-commerce training. Regarding their degree of knowledge of e-commerce, only 25.8% of the main body was relatively knowledgeable, and about 50% had an average understanding of e-commerce. In terms of household labor, the vast majority of e-commerce households had a household labor force greater than one person, which indicates that household livelihoods do not depend on one person and that life pressures are shared. In terms of main business, agricultural products and agricultural by-products predominate, with 46.9% of agricultural products being sold and 29.2% of agricultural by-products being processed and sold.

### 3.2. Selection of Variables

(1a) Latent variable: willingness to upgrade. According to Ajzen’s research, combined with the domestic and foreign willingness survey scales and research on the theme of the digital upgrading of rural e-commerce, we set “Q1: rural e-commerce households’ willingness to upgrade digitally”; “Q2: rural e-commerce households’ way of using big data (establish big data thinking, improve the ability of data collection, mining and analysis; Transform the management mode of e-commerce business; optimize the professional talent team, improve the ability of data collection, mining and analysis; strengthen the construction of database; strengthen the stability of data platform; improve the level of risk management to ensure the security of their own and customers’ information”; “Q3: Whether to receive training on e-commerce business”; “Q4: Whether training and performance assessment are provided for marketing staff”; “Q5: Choice of digital sales methods (short video, live streaming, public number, small program, fan group, micro business, soft promotion, others)”; and “Q6: adoption of big data or not” as six measurable variables.

(1b) Latent variable: behavioral attitude. Based on the research results of Ajzen, Li, Wenlong, KIM E, and Wu, Dan, this paper designed four measurable variables: “Q7: online sales share”; “Q8: product platform competitiveness”; “Q9: digital upgrade understanding”; and “Q10: online revenue share”. Meanwhile, based on the theory of planned behavior, “Q11: Sales growth after operating e-commerce”, “Q12: Revenue growth after operating e-commerce”, “Q13: Sales after digital upgrade”, and “Q14: Sales after digital upgrade” were designed from the perspective of behavioral outcome assessment. “Q15: Average annual income in the last three years”, “Q16: Product acquisition”, and “Q17: Income after digital upgrade” were designed from the perspective of the strength of behavioral beliefs. “Q16: Product acquisition (acquisition by individual rural households; cooperative production; base production; independent company production; home production; procurement from other regional suppliers; others)” and “Q17: Third-party platform diversion methods (WeChat; Weibo; Xiaohongshu; Jieyin; Faster; Beili Beili; Volcano Video; Watermelon Video; Tencent Weishi; Today Headlines; Meipai; VWAP; Ctrip; Where to go; Others)” and “3 measurable variables” were also used.

(2) Latent variable: subjective norms. According to the findings of scholars such as Yan Beibei, Mu J, Wang Peng and Chen X, this paper designed “Q18: Number of household participation in e-commerce”; “Q19: Perception of e-commerce training services”; “Q20: the improvement of e-commerce logistics facilities”; “Q21: the construction status of e-commerce service centers”; “Q22: the number of e-commerce practitioners in this village”; and “Q23: the proportion of e-commerce income in the village”. Meanwhile, based on the theory of planned behavior, “Q24: the proportion of online transactions of local village e-merchants”; “Q25: participation in e-merchant associations”; “Q26: government measures to support rural e-merchants from the perspective of normative beliefs (tax incentives; intellectual property protection policies; special fund support or policy financing guarantee support; government subsidies; intellectual support such as government technical training and talent introduction; government purchase; economic public services; leading organization of e-commerce associations; strengthening market supervision; leading enterprises involved in rural e-commerce)”; “Q27: Q28: Type of e-commerce business”; “Q29: Whether or not a brand has been created”; “Q30: Product marketing methods (e.g., the number of people in the market)”; and “Q31: The number of people in the market”. “Q30: Product marketing methods (traditional advertising methods; inviting internet celebrities to increase traffic and popularity; live-streaming by the store itself; creating special publicity for their own products; discount promotions; changing marketing methods according to competitors’ marketing strategies; event marketing; strengthening the creation of quality content for their own products to harvest more target people; others)” and “Q31: Ways to maintain relationships with fans (build a database of fan resources; treat fans as friends; fan benefits; produce content according to fans’ needs; deliver positive energy; interact with fans regularly; others)” were two of the four measurable variables.

(3) Latent variable: perceptual behavioral control. Based on the findings of scholars such as Mican D, Yang Shoude, Xiao H and Li Yuanjun, this paper designed “Q32:Product sales channels (community e-commerce; third-party e-commerce platforms; self-media platforms; social platforms; cross-border e-commerce; others)”, “Q33:Digital sales methods of e-commerce in this village (short video; live broadcast; public number; fan group; small program; micro business; soft promotion; others)”, and “Q 34:Methods to enhance the added value of products in this village (agricultural products have differentiated selling points; agricultural products have unique images, such as packaging; agricultural products deep processing; promotion at exhibitions, agricultural fairs and national agricultural fairs; cultural and tourism e-commerce; products with obvious brand advantages; others)”, as well as another three measurable variables. Meanwhile, according to the theory of planned behavior, “Q35:Literacy level of rural e-commerce households”, “Q36: Number of rural e-commerce households’ labor force”, “Q37: Difficulties encountered in digital upgrading (lack of experience and management skills; lack of capital; increased costs due to new sales methods; lack of technical skills; lack of energy; lack of easy access to more sources; other)”, “Q38: Age of rural e-merchant households”, “Q39: Age of rural e-merchant households, “Q40: Number of times to start a business before running an e-commerce business”, “Q41: Degree of understanding of e-commerce before running an e-commerce business”, “Q42: Main business of e-commerce business (sales of agricultural products; processing and selling of agricultural and sideline products; and Sales of agricultural products; processing and selling of agricultural and sideline products; processing and selling of furniture and timber; selling of small commodities; selling of textiles and garments; farming and rural tourism; selling of agricultural production materials; others)”, and “Q43:Convenience of nearby transportation” were designed from the perspective of perceptual strength. “Q44: Difficulties encountered in the business process (product quality and standards are not up to standard and difficult to sell online; products have a short shelf life and are prone to damage and deterioration in transit; imperfect rural logistics infrastructure; high logistics costs; high operating costs of e-commerce platforms; not finding a suitable marketing method for products; products without characteristics and too many products of the same type in the market; others)”, “Q45: Direct road condition”“, “Q46: Proximity to station”, and “Q47: Proximity to courier point” are the five measurable variables.

### 3.3. Research Methodology

This paper constructs a hypothetical model of rural e-commerce digital upgrading intentions based on Ajzen’s theory of planned behavior, which focuses on the links between latent variables such as rural e-commerce behavioral attitudes, subjective norms, perceived behavioral control and rural e-commerce digital upgrading intentions. Latent variables are generally not directly observable, but they can be measured by measuring some exogenous indicators to respond to these latent variables. Structural equation modeling can deal with latent variables and their associated exogenous indicators, while allowing for measurement errors between the independent and dependent variables, as well as greater elasticity of measurement models and estimations of model fit. Therefore, this paper uses structural equation modeling to analyze the willingness to digitally upgrade rural e-commerce and its influencing factors, using the following structural and measurement equations.

Structural equation model:

η = Bη + Γξ + ζ(1)

Measurement equation model:

(2) $$y=\Lambda_{y}\eta +\varepsilon$$

(3) $$x=\Lambda_{x}\xi +\delta$$

In Equation (1), B is the path coefficient, indicating the relationship between endogenous latent variables; ƞ is the endogenous latent variable, indicating the willingness to digitally upgrade rural e-commerce, behavioral attitudes, subjective norms, and perceptual behavioral control; Γ indicates the effect of the exogenous latent variable ξ on the endogenous latent variable; and ζ is the residual term, indicating the part of the equation that cannot be explained. In Equations (2) and (3), X denotes the observable variable of the exogenous latent variable; Y denotes the observable variable of the endogenous latent variable; $\Lambda_{x}\;$denotes the direct relationship between the exogenous observed variable and the exogenous latent variable, and is the factor loading matrix of the exogenous observed variable on the exogenous latent variable;$\;\Lambda_{y}\;$denotes the relationship between the endogenous observed variable and the endogenous latent variable, and is the factor-loading matrix of the endogenous observed variable on the endogenous latent variable; and ξ and δ denote the error. In the structural equations of this paper, endogenous latent variables denote willingness to digitally upgrade rural e-commerce, behavioral attitudes, subjective norms and perceptual behavioral control, and exogenous latent variables include behavioral belief strength, outcome assessment, normative beliefs, compliance motivation, control beliefs and perceptual strength.

## 4. Analysis of Results

### 4.1. Reliability and Validity Tests of the Questionnaire

(1) Reliability test of the data. First, factor rotation analysis was conducted in SPSS 24.0 using the maximum variance method on 47 indicators. Eleven indicators with factor loading coefficients below 0.5 were excluded [46], and then Bartlett’s spherical test was conducted on the remaining thirty-six indicators, with a KMO value of 0.674, and Bartlett’s spherical test result was significant at the *p* = 0.000 level, indicating that the scale has a good correlation. Finally, the remaining 36 indicators were analyzed for reliability and the Cronbach’s alpha values were all greater than 0.6 (see Table 2), indicating that the scale has reliable reliability [47].

(2) Validity test of the data. The latent variable path conception and questionnaire setting of this questionnaire are the result of a comprehensive consideration based on relevant theoretical studies, a literature review and pre-research data, so the questionnaire has good content validity and criterion validity. As shown in Table 3, the factor loading coefficients of each scale were greater than 0.5 and met the significance requirements of *p* < 0.05, which indicated that the data had high validity and the latent variables willingness to upgrade, behavioral attitudes, subjective norms, perceived behavioral control, behavioral belief strength, outcome assessment, normative beliefs, motivation to conform, control beliefs, and perceived strength were all interpreted by their corresponding measurable variables.

### 4.2. Model Fitting Results

**Figure 2:**
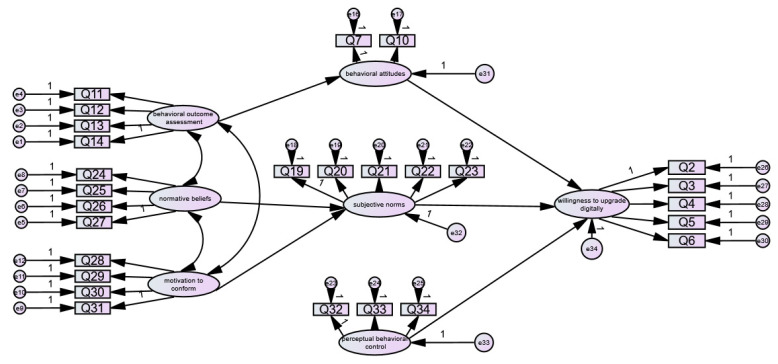
Initial path of willingness to digitally upgrade rural e-commerce.

According to the hypothetical model and data of willingness to digitally upgrade rural e-commerce, the structural equation was fitted using the Amos 28.0 software, and it was found that the latent variable behavioral belief intensity could not be successfully fitted; the reason for this could be that it contains only two question terms and H2 does not hold. After removing the path from behavioral belief strength to behavioral attitude and fitting again, it was found that the coefficients of the path from perceptual strength and control belief to perceptual behavior control were not significant, and H8 and H9 were not valid, so the path from perceptual strength and control belief to perceptual behavior control was removed to obtain Figure 2.

According to Figure 2, the initial model-fit values were obtained by running Amos 28.0, and the fitting results and evaluation criteria are shown in Table 4.

### 4.3. Revision and Finalization of the Model

(1) Revision of the model. Since the above model fitting results are poor and the model fitting goodness should be improved, the hypothetical model of digital upgrading of rural e-commerce was amended. Because the sample data were tested for scientificity and passed the reliability and validity tests, the measurable indicators of the latent variables were not modified. With reference to the experience of previous scholars, the covariance correction index was amended, combining the actual research situation and the principle of adjusting one parameter at a time [48,49]. During the amendment process, coefficients of the path from perceptual behavior control to upgrade willingness changed from significant to insignificant, so the path was deleted. The specific correction was to change the residuals e1 and e2, e5 and e20, e19 and e20, e8 and e18, e6 and e32, e6 and e18, e6 and e34, e6 and e20, e20 and e31, e26 and e28, e1 and e29, e6 and e30, e9 and e29, e11 and e31, e6 and e20, e5 with e18, e17 with e22, e26 with e27, and e10 with e27; these were connected.

(2) Optimal results after model modification. As can be seen from Table 5, all the fit indices were substantially optimized, CMIN/DF was significantly reduced, GFI, IFI and CFI all reached the ideal standard, RMSEA reached an acceptable range, and the values of other indicators were also good, which shows the good fit of the modified model of the willingness to digitally upgrade rural e-commerce.

The sample data were normalized to obtain the statistics of the latent variable effects of the optimized model and the coefficients of each path of the optimized model. As can be seen from the values in Table 6, the optimized model path coefficients were all significant.

### 4.4. Analysis of Results

From the above analysis, the hypothetical model of rural e-commerce digital upgrade willingness was validated. Both behavioral attitudes and subjective norms of rural e-merchants have significant effects on the willingness to upgrade, where there is a positive effect of behavioral attitudes on willingness to upgrade, with a path coefficient of 0.329, and a negative effect of subjective norms on willingness to upgrade, with a path coefficient of −0.047, meaning that H1 is valid and H4 is not valid. This indicates that, in the model of rural e-commerce digital upgrade willingness, rural e-commerce behavioral attitudes have the greatest effect on upgrade willingness and subjective norms have a slightly smaller effect. Table 6 also reveals that behavioral outcome assessments, compliance motivation and normative beliefs have an indirect effect on rural e-commerce upgrading intentions, with behavioral outcome assessment (0.047) having the largest effect, followed by compliance motivation (0.004), and normative beliefs (−0.031), which have a negative effect on upgrading intentions.

(1) Rural e-commerce behavioral attitudes have a positive impact on willingness to digitally upgrade. As can be seen from Table 7, the two observed variables of rural e-merchants’ behavioral attitudes, the share of online sales (Q7) and the share of online revenue (Q10), are both significant at the 0.001 level, and their path coefficients are 0.876 and 0.501, respectively. This indicates that, in the formation of rural e-merchants’ willingness to digitally upgrade, their behavioral attitudes are objectively influenced by the joint effect of Q7 and Q10, and the vast majority of e-merchants consider the proportion of online and offline sales to be the most important factor influencing their behavioral attitudes towards digital upgrades. The reason for this is that rural e-merchants’ income drives their behavioral attitudes, and all their behaviors are aimed at increasing their income; the larger the proportion of online sales and online income of rural e-merchants, the better their behavioral attitudes towards digital upgrades.

Among the latent variables affecting behavioral attitudes towards rural e-commerce, the path coefficient of behavioral outcome assessment is 0.142 and is significant at the 0.001 level, so it can prove that hypothesis H3 holds. From Table 7, it can be seen that all the observed indicators of behavioral outcome assessment are 0.982, 0.922, 0.484, and 0.296 and all are significant at the 0.001 level, which indicates that the four observed variables of sales growth after operating e-commerce (Q11), income growth after operating e-commerce (Q12), sales after digital upgrade (Q13) and income after digital upgrade (Q14) have a significant positive effect and significantly act on willingness to upgrade through behavioral attitudes. Sales volume growth after operating an e-commerce business and income growth after operating an e-commerce business had the deepest impact on the behavioral outcome assessment of rural e-commerce. The reason for this is that growth in sales volume and growth in income were the most important factors in the outcome assessment of rural e-commerce households.

(2) Subjective norms of rural e-commerce have a significant negative effect on the willingness to digitally upgrade. As can be seen from Table 7, the path coefficients of four observations of subjective norms are greater than 0.5, and the path coefficient of another indicator is also close to 0.4 and significant at the level of 0.1, indicating that the perception of e-commerce training services (Q19), the state of improvement in e-commerce logistics facilities (Q20), the state of construction of e-commerce service centers (Q21), the number of e-commerce employees in this village (Q22) and the percentage of e-commerce income in this village (Q23) act on the subjective norms of rural e-merchants, which affect the intention to digitally upgrade. This suggests that the worse the rural e-commerce experience, the less convenient the logistics, the less well-developed the local support services, and the lower the number of people engaged in e-commerce, the weaker their subjective norms will be, which leads to weak willingness to digitally upgrade rural e-commerce.

Among the latent variables affecting the subjective norms of rural e-commerce, the path coefficient of normative beliefs (0.666) and the path coefficient of obedience motivation (−0.088) is significant at the 0.001 level, thus verifying H5 and H6. This indicates that, in terms of subjective norms, normative beliefs are the most important factor affecting the subjective norms of rural e-commerce. Meanwhile, as shown in Table 7, the path coefficients of the percentage of online transactions of e-commerce in this village (Q24), participation in e-commerce associations (Q25), government measures to support rural e-commerce (Q26) and the improvements in e-commerce data information center (Q27) on subjective norms are 0.786, 0.800, 0.542 and 0.750, respectively, indicating that all four indicators play a significant role in normative beliefs and significantly affect the willingness to digitally upgrade rural e-commerce through subjective norms, with local participation in e-commerce associations having the greatest impact. As can be seen in the above table, the path coefficients of the type of e-commerce business (Q28), whether or not a brand is created (Q29), product marketing methods (Q30), and the means of maintaining relationships with fans (Q31) indicators for compliance motivation are 0.358, 0.624, 0.825, and 0.377, respectively, which indicates that these four indicators influence rural e-merchants’ subjective norms through compliance motivation, which, in turn, influences their willingness to upgrade, with product marketing methods having the greatest impact. The hypotheses presented in the paper were verified or not as shown in Table 8.

## 5. Research Findings and Policy Implications

### 5.1. Findings of the Study

This paper takes 1387 rural e-commerce households in Zhejiang Province as a sample. Based on the theory of planned behavior, we used the SPSS24.0 and Amos28.0 software to conduct an empirical analysis of the willingness to digitally upgrade rural e-commerce and the influencing factors, verify the influence of behavioral attitudes and subjective norms on the willingness to digitally upgrade rural e-commerce, and indirectly explore behavioral outcome assessments, behavioral belief strength, normative beliefs, conformity motivation, control beliefs and perceptual strength regarding rural e-commerce digital upgrade willingness. We then aimed to reveal the formation mechanism of rural e-commerce digital upgrade willingness from a holistic perspective. The specific findings are as follows. First, the behavioral attitudes of rural e-commerce households have a positive and significant impact on the willingness to digitally upgrade, while normative beliefs have a negative and significant impact on willingness to digitally upgrade, with the behavioral attitudes of rural e-commerce being more influential than their normative beliefs. Second, in terms of behavioral attitudes, the higher the proportion of online sales or the proportion of online income sources of rural e-commerce households, the more the behavioral attitudes promote the digital upgrading of rural e-commerce households. Third, in terms of subjective norms, when rural e-commerce households perceive that the number of participants in their village’s e-commerce association is too high and e-commerce income is saturating the village’s total income, indicating that competition is in a fierce state, the subjective norms constructed on this basis have a suppressive effect on rural e-commerce digital upgrading willingness.

### 5.2. Policy Implications

Based on the above findings, the willingness of rural e-merchants to digitally upgrade can be enhanced in two ways: (1) by increasing sales and income through improving the degree of digital upgrading, thus fostering positive behavioral attitudes; (2) by guiding e-merchants in villages to correctly participate in the digital upgrading of e-commerce associations, not to blindly follow trends, and to avoid homogeneous competition, thus reducing the negative influence of subjective norms.

In response to the problem of inactive behavioral attitudes of rural e-commerce, the government can take measures in terms of behavioral outcome assessment, focusing on improving the online sales and income of rural e-commerce households. First, the government’s service mechanism for rural e-commerce households could be improved to guide farmers to operate e-commerce as well as digitally upgrade, build a more scientific mechanism for rural e-commerce operation and digital upgrades, lower the threshold for farmers to operate, and improve rural e-commerce online sales. Secondly, using the theory of rural revitalization as a guide, the mechanisms by which farmers’ income could be improved, and tax or rent relief could be provided via online commodity sales to improve farmers’ income.

To address the negative impact that subjective norms have on rural e-commerce, the government should reduce the negative impact of control beliefs and obedience motives on rural e-commerce. In response to the control beliefs of rural e-commerce, the government should improve the online trading mechanism of e-commerce, reduce the in-roll of local rural e-commerce, avoid the grouping of e-commerce associations to squeeze the profits of other individual e-commerce, increase the support for rural e-commerce, and ensure that data and information construction keep up with the development of rural e-commerce. In response to the obedience motive of rural e-merchants, the government should advocate that farmers choose their own type of business, instead of just following the trend, improving their brand creation mechanism, encourage rural e-merchants to create their own brands, help them to try more marketing methods, and strengthen the connection with their fans.

### 5.3. Research Contributions and Perspectives

Afful-Dadzie’s study on the motivation of ICT adoption in rural communities, Alibaygi’s study on the positive effects of ICT, Ye’s case study on ICT and Schwering’s study on farmers’ willingness to use rural e-commerce mainly analyze the adoption of digitalization in rural communities from extrinsic, intrinsic and mixed motivations, without making a careful division of these influencing factors and without launching an analysis of their specific causes from the perspective of behavioral science, so this paper explores the behavioral motives of digital upgrading of rural e-commerce based on the former study and the theory of planned behavior, and the findings of this paper show that human behavioral attitudes and subjective norms are the most important factors influencing the willingness of digital upgrading of rural e-commerce. The research in this paper will help the government to take targeted measures to enhance willingness regarding rural e-commerce digital upgrades, reduce unnecessary expenditures, and improve the efficiency of rural e-commerce digital upgrades. However, there are limitations in this paper, as the influence of other national and international factors on willingness to digitally upgrade rural e-commerce was not considered, so scholars can study the willingness to digitally upgrade from the perspective of other provinces or countries in the future and compare these with this paper to explore the similarities and differences.

## Figures and Tables

**Table 1:** Overview of the rural e-commerce sample.

Features	Detailed Classification	Percentage (%)	Features	Detailed Classification	Percentage (%)
age	Under 30 years of age	18.7%	educational level	Primary school and below	8.5%
	30–39 years	38.1%		Junior high school	19.3%
	40–49 years	33.2%		High school	21.0%
	50–59 years	8.2%		Post-secondary	26.4%
	60 years and over	1.7%		Undergraduate (adjective)	23.4%
Number of business start-ups	0	52.3%		Postgraduate and above	1.4%
	1	33.2%	Level of understanding of e-commerce	Not at all.	3.1%
	2	10.7%		Relatively unaware	20.3%
	3 times or more	3.8%		General	50.1%
Number of training sessions on e-commerce operations	0	57.0%		Relatively familiar	25.8%
	1	9.7%		Very knowledgeable.	0.6%
	2	15.1%	Main business (multiple options available)	Agricultural products’ sales	46.9%
	3	10.8%		Processing and sales of agricultural and sideline products	29.2%
	4	2.8%		Furniture woodworking sales	7.7%
	5 times and above	4.6%		Sales of small goods	16.9%
Number of people in the labor force	1	4.1%		Sales of textiles and clothing	19.8%
	2	43.3%		Nongkak, rural tourism	15.4%
	3	30.4%		Sale of agricultural production materials	6.3%
	4	15.2%		Other	7.7%
	5	7.0%			

**Table 2:** Reliability tests for latent variables.

Latent Variable	Cronbach’s Alpha	Number of Variables
Willingness to upgrade	0.681	5
behavioral attitude	0.615	2
Assessment of behavioral outcomes	0.784	4
Strength of behavioral beliefs	0.678	2
Subjective norm	0.766	5
Normative beliefs	0.781	4
obedience-motivated	0.603	4
Perceptual Behavioral Control	0.655	3
Control beliefs	0.634	3
Perceptual intensity	0.701	4

**Table 3:** Variable validity tests.

Latent Variable	Measurable Variables	Factor Load	Average Value	Standard Error	*p*-Value
Willingness to upgrade	Q2	0.737	2.03	0.029	0.000
	Q3	0.653	1.65	0.029	0.000
	Q4	0.557	0.31	0.012	0.000
	Q5	0.593	2.20	0.027	0.000
	Q6	0.700	0.36	0.013	0.000
Behavioral attitude	Q7	0.769	3.04	0.028	0.000
	Q10	0.745	2.47	0.030	0.000
Assessment of behavioral outcomes	Q11	0.890	2.93	0.007	0.000
	Q12	0.881	2.91	0.008	0.000
	Q13	0.741	2.94	0.007	0.000
	Q14	0.595	2.90	0.009	0.000
Strength of behavioral beliefs	Q15	0.811	2.69	0.030	0.000
	Q16	0.814	1.45	0.018	0.000
subjective norm	Q19	0.642	2.72	0.033	0.000
	Q20	0.668	3.81	0.017	0.000
	Q21	0.613	3.39	0.024	0.000
	Q22	0.804	2.23	0.030	0.000
	Q23	0.819	2.25	0.029	0.000
Normative beliefs	Q24	0.658	2.96	0.031	0.000
	Q25	0.699	2.21	0.022	0.000
	Q26	0.826	3.81	0.028	0.000
	Q27	0.594	3.40	0.025	0.000
Obedience-motivated	Q28	0.580	1.17	0.014	0.000
	Q29	0.683	0.30	0.012	0.000
	Q30	0.783	2.42	0.026	0.000
	Q31	0.594	2.18	0.025	0.000
Perceptual Behavioral Control	Q32	0.739	2.75	0.027	0.000
	Q33	0.813	3.40	0.038	0.000
	Q34	0.710	2.13	0.029	0.000
Control beliefs	Q35	0.717	2.84	0.028	0.000
	Q37	0.676	2.55	0.023	0.000
	Q38	0.725	2.79	0.025	0.000
Perceptual intensity	Q43	0.691	4.29	0.019	0.000
	Q45	0.688	2.68	0.014	0.000
	Q46	0.740	2.49	0.016	0.000
	Q47	0.725	2.43	0.016	0.000

**Table 4:** Evaluation index system and fitting results of the overall fitness of the structural equations.

Index Name		Evaluation Criteria	Initial Fitted Value	Result
Absolute fit index	CMIN/DF	1–3 is best	16.720	no good
	GFI ^1^	Greater than 0.9	0.789	no good
	RMSEA ^2^	Less than 0.05 is better, less than 0.08 is fair	0.106	no good
	ECVI	Less than saturated model and independent model values	3.891	ideals
	NFI ^3^	Greater than 0.9	0.658	no good
	IFI ^4^	Greater than 0.9	0.672	no good
Relative fit index	TLI ^5^	Greater than 0.9	0.633	no good
	CFI ^6^	Greater than 0.9	0.671	no good
	AIC ^7^	The smaller the better.	5392.884	no good
Information index	PNFI	Greater than 0.5	0.591	ideals
	PCFI	Greater than 0.5	0.602	ideals

^1^ Goodness of Fit Index. ^2^ Root Mean Square Error of Approximation. ^3^ Normative Fit Index. ^4^ Incremental Fit Index. ^5^ Tucker–Lewis Index. ^6^ Comparative Fit Index. ^7^ Bare Pool Information Criterion.

**Table 5:** Calculated results of the modified model fit index.

Targets	CMIN/DF	GFI	RMSEA	ECVI	NFI	IFI	TLI	CFI	AIC	PNFI	PCFI
result	6.870	0.915	0.065	1.223	0.885	0.900	0.877	0.900	1695.730	0.721	0.733

**Table 6:** Results of direct effects, indirect effects, and standardization of total effects among the potential variables in the model.

Variable	Effect (Scientific Phenomenon)	Obedience-Motivated	Normative Beliefs	Assessment of Behavioral Outcomes	Subjective Norm	Behavioral Attitude	Willingness to Upgrade
Subjective norm	Aggregate effect	−0.088	0.666	0	0	0	0
	Direct effect	−0.088	0.666	0	0	0	0
	Indirect effect	0	0	0	0	0	0
Behavioral attitude	Aggregate effect	0	0	0.142	0	0	0
	Direct effect	0	0	0.142	0	0	0
	Indirect effect	0	0	0	0	0	0
Willingness to upgrade	Aggregate effect	0.004	−0.031	0.047	−0.047	0.329	0
	Direct effect	0	0	0	−0.047	0.329	0
	Indirect effect	0.004	−0.031	0.047	0	0	0

**Table 7:** Estimated normalized path coefficients for the optimization model.

Path Relation	Path Coefficient	Standard Error	Critical Ratio	*p*
Q2 <-- Willingness to upgrade	0.876			
Q3 <-- Willingness to upgrade	0.609	0.041	17.148	<0.001
Q4 <-- Willingness to upgrade	0.604	0.018	16.650	<0.001
Q5 <-- Willingness to upgrade	0.450	0.034	14.017	<0.001
Q6 <-- Willingness to upgrade	0.622	0.019	17.015	<0.001
Willingness to upgrade <-- Behavioral attitudes	0.329	0.040	8.520	<0.001
Q7 <-- Behavioral attitudes	0.876			
Q10 <-- Behavioral attitudes	0.501	0.060	10.200	<0.001
Behavioral attitudes <- Assessment of behavioral outcomes	0.142	0.279	4.479	<0.001
Q11 <-- Behavioral outcome assessment	0.982	0.233	11.149	<0.001
Q12 <-- Behavioral outcome assessment	0.922	0.230	11.357	<0.001
Q13 <-- Behavioral outcome assessment	0.484	0.083	14.153	<0.001
Q14 <-- Behavioral outcome assessment	0.296			
Willingness to upgrade <-- subjective norm	−0.047	0.040	−1.672	0.095
Q19 <-- Subjective norms	0.519			
Q20 <-- Subjective norms	0.509	0.033	15.081	<0.001
Q21<-- Subjective norms	0.388	0.041	12.671	<0.001
Q22 <-- Subjective norms	0.747	0.066	19.125	<0.001
Q23 <-- Subjective norms	0.923	0.076	19.795	<0.001
Subjective norms <- normative beliefs	0.666	0.038	16.631	<0.001
Q24 <-- Normative beliefs	0.786	0.047	28.672	<0.001
Q25 <-- Normative beliefs	0.800	0.031	30.383	<0.001
Q26 <-- Normative beliefs	0.542	0.044	18.881	<0.001
Q27 <-- Normative beliefs	0.750			
Subjective norms <-- Motivation for obedience	−0.088	0.052	−3.292	<0.001
Q28<-- Motivation for obedience	0.358	0.060	8.905	<0.001
Q29<-- Motivation for obedience	0.624	0.074	11.221	<0.001
Q30<-- Motivation for obedience	0.825	0.217	10.975	<0.001
Q31<-- Motivation for obedience	0.377			

**Table 8:** Hypothesis confirmation and non-confirmation table.

Assumptions	Whether It Is Confirmed
H1	Yes
H2	Not proven but useful
H3	Yes
H4	Not proven but useful
H5	Yes
H6	Yes
H7	Not proven but useful
H8	Not proven but useful
H9	Not proven but useful

## Data Availability

Not applicable.
